# Epitranscriptomic and expression profiling in *Riccia fluitans* across diverse environmental conditions

**DOI:** 10.1016/j.csbj.2025.10.002

**Published:** 2025-10-02

**Authors:** Mateusz Maździarz, Katarzyna Krawczyk, Joanna Szablińska-Piernik, Łukasz Paukszto, Monika Szczecińska, Paweł Sulima, Jakub Sawicki

**Affiliations:** a Department of Botany and Evolutionary Ecology, University of Warmia and Mazury in Olsztyn, Plac Łódzki 1, Olsztyn 10-719, Poland; b Department of Genetics, Plant Breeding and Bioresource Engineering, University of Warmia and Mazury in Olsztyn, Plac Łódzki 3, Olsztyn 10-724, Poland

**Keywords:** Riccia fluitans, Epitranscriptome, N6-methyladenosine, 5-methylcytosine, Pseudouridine, Expression profiling, Metabolites

## Abstract

Post-transcriptional RNA modifications, such as N6-methyladenosine (m6A), 5-methylcytosine (m5C), and pseudouridine (Ψ), are critical regulators of plant development, stress responses, and environmental adaptation. The roles of m6A, m5C, and Ψ in plant stress responses have been insufficiently explored. To investigate the epitranscriptomic landscape of these modifications, we employed direct RNA sequencing (DRS) to analyze native RNA landscape from the amphibious plant *Riccia fluitans* grown in both aquatic and terrestrial environments. Our study revealed the presence of Ψ, m5C, and m6A modifications and expression profiling in *R. fluitans* transcriptomes from diverse environments. Metabolomic profiling further demonstrated environment-dependent shifts in soluble sugars, aligning with transcriptional and epitranscriptomic changes in starch metabolism genes. Collectively, our findings highlight the dynamic regulation of RNA modifications as a crucial and previously underappreciated component of environmental adaptation in early land plants, providing new insights into the molecular basis of phenotypic plasticity and the evolutionary transition to terrestrial life.

## Graphical Abstract

**Figure:**
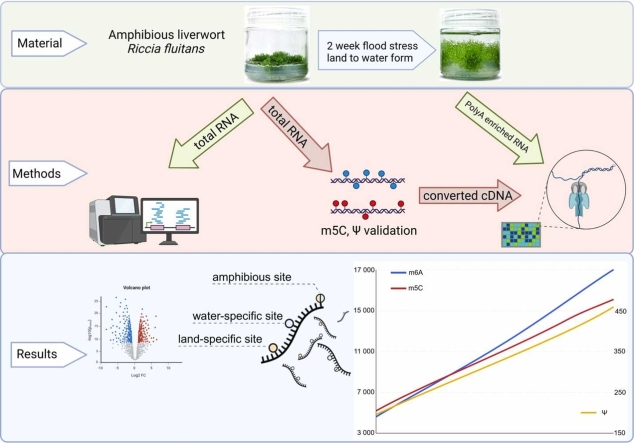

**Key Message**

Direct RNA sequencing uncovers the role of post-transcriptional RNA modifications (m6A, m5C, and Ψ), revealing their significant importance in the environmental adaptation and stress responses of amphibious plants in aquatic and terrestrial habitats.

## 1 Introduction

Post-transcriptional RNA modifications, such as N6-methyladenosine (m6A), 5-methylcytosine (m5C), and pseudouridine (Ψ), play crucial roles in plant development, stress responses, and adaptation to changing environments [10], [25], [3], [52], [53], [7], [70]. Among these modifications, m6A is the most abundant internal mRNA modification in eukaryotes [52]. It regulates RNA splicing, export, stability, and translation, thereby influencing key developmental processes such as flowering, root formation, and embryogenesis [4]. The dynamic and reversible nature of m6A allows plants to fine-tune gene expression in response to various stresses, including drought, temperature extremes, salinity, and pathogen attack [21], [24], [3], [34], [61]. While m6A dominates the epitranscriptome landscape, m5C and Ψ also contribute substantially to RNA regulation in plants. The m5C modification, which involves methylation at the carbon-5 position of cytosine, occurs in mRNA, rRNA, and tRNA [48], [61]. It has been detected in *Arabidopsis thaliana* and *Oryza sativa*, where it affects plant development, stress tolerance, and long-distance mRNA transport [12], [18], [62], [68]. For example, OsNSUN2 mutants in rice exhibit reduced root length and increased heat sensitivity due to impaired methylation of stress-related transcripts [53]. Ψ, resulting from uridine isomerization, enhances RNA stability and structure [38]. Although less studied in plants, Ψ modifications have been linked to seed germination, root growth, and flowering, suggesting similar roles in mRNA metabolism as observed in animals [36], [48], [50], [52]. RNA modifications such as m6A, m5C, and Ψ are typically detected using antibody-based or chemical methods, including MeRIP-seq for m6A and bisulfite sequencing for m5C, though these approaches can suffer from biases and limited resolution [10], [52], [7]. Ψ is often identified through chemical modification (e.g., CMCT treatment) or mass spectrometry, yet most existing methods require parallel controls and cannot detect multiple modifications simultaneously [39], [8]. These limitations have driven growing interest in direct RNA sequencing (DRS) using nanopore technology, which enables the simultaneous detection of multiple RNA modifications without the need for reverse transcription, amplification, or chemical labeling.

Direct RNA sequencing (DRS) with nanopore technology offers a powerful alternative for epitranscriptome analysis, overcoming many limitations of traditional approaches. Unlike short-read sequencing platforms that rely on reverse transcription and PCR amplification, DRS sequences native RNA molecules directly, preserving modification signatures and allowing for their detection in full-length transcripts [16], [57]. This method enables the simultaneous analysis of multiple RNA modifications, including m6A, m5C, and Ψ, by detecting characteristic disruptions in the ionic current as the RNA strand translocates through the nanopore [1], [36]. Under appropriately trained models and sufficient read depth, DRS can detect RNA modifications at base resolution in individual reads while preserving the transcript’s native structure and isoform-specific modification patterns. However, detection accuracy depends on model training, calibration, and coverage, and for Ψ and m5C on R9.4.1 currently provides only moderate confidence. Moreover, DRS captures transcript isoforms, poly(A) tail lengths, RNA structure, and abundance in a single experiment, offering a comprehensive view of transcriptome regulation [42], [5]. Additionally, because DRS does not require antibody enrichment, chemical derivatization, or fragmentation, it reduces experimental biases and streamlines sample preparation. It is particularly advantageous for species with incomplete genome annotations or without well-characterized antibodies for specific RNA modifications. Furthermore, the continuous improvements in basecalling algorithms and machine learning models have significantly enhanced the accuracy of modification detection from raw nanopore signal data [44], [64]. As such, DRS has become a promising tool for exploring the complexity and dynamics of the plant epitranscriptome and is especially valuable for non-model organisms with sparse genomic resources [28], [36]. DRS provides a unique opportunity to explore transcriptomic and epitranscriptomic adaptations in non-model organisms such as amphibious plants, which thrive in fluctuating environments [2], [29]. The aquatic liverwort *Riccia fluitans* serves as an excellent model for studying these adaptations due to its remarkable developmental plasticity. It dynamically adjusts its morphology and physiology to either aquatic or terrestrial conditions—producing thin tissues to facilitate gas exchange when submerged, and thicker, starch-accumulating tissues when exposed to air [2]. Despite their ecological significance, amphibious plants—particularly non-vascular representatives like *R. fluitans*—remain underexplored regarding epitranscriptomic regulation.

We hypothesize that RNA modifications contribute to environmental adaptation in *R. fluitans*. To test this, we investigated how shifts between aquatic and terrestrial habitats reshape the RNA modification landscape in *R. fluitans*. Specifically, we focused on three major modifications—m6A, m5C, and Ψ—and assessed their dynamic regulation during habitat transition. By applying direct RNA sequencing (DRS), we conducted transcriptome-wide analyses to detect base-resolution modification changes in individual reads while preserving isoform-specific patterns. To enhance detection of cytosine and uridine modifications, we adapted the PRAISE method [67] for nanopore cDNA sequencing. Our analysis centered on differentially modified transcripts linked to key adaptive processes, including water homeostasis, starch metabolism, and cell wall remodeling. By integrating modification profiles with gene expression and metabolite measurements, we sought to elucidate the role of RNA modifications in the ecological versatility of *R. fluitans.*

## 2 Materials and methods

### 2.1 Plant material, RNA extraction and RNA sequencing

Plant material (*R. fluitans* RF1 line) was obtained from an established axenic *in vitro* culture [40], [47]. Cultures were maintained in climate chambers at 24°C under long-day conditions (16-hour light/8-hour dark). After four weeks, one set of cultures was submerged in sterile distilled water, while another set remained unchanged. This experimental setup was replicated four times and continued for two weeks. Total RNA was extracted with the RNA Plant Mini Spin kit (Qiagen) following the manufacturer's instructions. RNA quality and quantity were assessed using a Tapestation (Agilent) with the High Sensitivity RNA Screening Tape kit and a Qubit 4 fluorometer with the HS RNA Assay Kit.

Long-read native RNA libraries were prepared from 50 ng of poly(A)-tailed mRNA per sample using the Direct RNA Sequencing Kit SQK-RNA002 (Oxford Nanopore Technologies). Ribosomal RNA was removed using NEBNext Poly(A) mRNA Magnetic Isolation Module (New England Biolabs). SuperScript III Reverse Transcriptase (Thermo Fisher Scientific) was used to synthesize the second strand of cDNA, forming RNA-cDNA hybrids. Sequencing adapters were then ligated using T4 DNA Ligase (New England Biolabs). Libraries were quantified with the Qubit dsDNA HS Assay Kit (ThermoFisher) and sequenced on a MinION MK1C device (ONT) with FLO-MIN 106 Flow Cells R.9.4.1 (ONT), prepared using the Flow Cell Priming Kit EXP-FLP002 (ONT). Raw reads were basecalled with Guppy v.6.0.0 [https://nanoporetech.com/platform/technology/basecalling]. The quality of the received FASTQ files was evaluated using FastQC software, and these files were subsequently utilized for further analyses. The data is available in the ENA EMBL-EBI database under accession numbers PRJEB72691 and PRJEB97949. Short-reads RNA sequencing libraries were constructed with the Truseq RNA library preparation kit (Illumina), incorporating the Ribo-Zero depletion step. Sequencing was performed on an Illumina NovaSeq 6000 platform at Macrogen Inc. (Seoul, Korea). Raw sequencing data has been deposited in the ENA EMBL-EBI database under accession number PRJEB72692.

### 2.2 Expression profiling based on short-reads

RNA quality assessment was performed using FastQC software [https://www.bioinformatics.babraham.ac.uk/projects/fastqc/]. Following RNA-Seq, Illumina adapters and poly-A segments were excised with the Trimmomatic tool v.0.39 [6] with these parameters: CROP:140 LEADING:20 TRAILING:20 MINLEN:140 AVGQUAL:20. Next, high-quality reads were mapped to the *Riccia fluitans* genome using the STAR v.2.7.11a tool [13] with the following parameters: --outFilterMultimapNmax 20 --outFilterMismatchNmax 999 --outFilterMismatchNoverLmax 0.04 --alignSJoverhangMin 8 --alignSJDBoverhangMin 1 --alignIntronMin 20 --alignIntronMax 1,000,000 --alignMatesGapMax 1,000,000. Obtained BAM files were used to create annotations with StringTie v.2.2.1 software [49]. StringTie aggregated individual GTF files from each sample and merged them into final annotations. Splicing variants of individual genes were obtained using the genomic annotations (GTF file). The count values for genes were then calculated by FeatureCounts v.2.0.6 with default parameters [33]. A statistical test (based on a negative model) implemented in the DESeq2 v.1.42.0 [37] R library was used to compare the expression profiles genes. Terrestrial samples were adopted as the reference condition. An absolute logarithmic fold change (log2FC) greater than 1 and an adjusted p-value (padj) less than 0.05 were set as cut-off values for significantly differentially expressed genes (DEGs).

### 2.3 Ψ detection

The transcriptome of *R. fluitans* was created using StringTie and gffread v.0.12.7 [43]. Information on Ψ was extracted from previously generated FASTQ files and transcriptome using the nanopsU program v.1.0.0 [26]. Next, gaps created during the read merging process were removed, and features from all U sites were extracted using scripts from the nanopsU program. Only U sites with over 20 reads were processed for further analysis. A Ψ region was considered reliable when the probability exceeded 0.8.

### 2.4 m5C detection

Information on m5C methylation was obtained using the CHEUI software [1]. Previously generated FASTQ files were mapped to the transcriptome sequence using the minimap2 software v.2.26 [31] with the -ax map-ont parameter. The resulting bam files were then sorted using samtools v.1.7.2 [11]. The signal data was rescaled to the aligned sequences using the nanopolish v.0.14.1 program [https://github.com/jts/nanopolish]. Preprocessing procedures assume two models: the first predicts modification within individual reads, second anticipates the stoichiometry and modification probability within transcriptomic sites. The RNA modifications that varied between terrestrial and aquatic environments were calculated using the CHEUI pipeline [https://github.com/comprna/CHEUI]. m5C modifications were classified as statistically significant when the probability exceeded 0.6. Sites present in both aquatic and terrestrial environments underwent differential analysis, where only those with pval_U < 0.05 and abs(stoichiometry diff) > 0.1 were deemed statistically significant.

### 2.5 m6A detection

The detection, significance, and differential analysis of m6A modifications were conducted using CHEUI software with the same parameters as those used in the m5C section. The CHEUI per-site detection results were compared to NanoSPA [26] and m6Anet v.2.1 [22]. To compare the results between these programs, it was established that all detected site adenine modifications with a probability exceeding 0.6 would be analyzed. All-context (AC) methylation was detected by the CHEUI package, while the NanoSPA and m6Anet packages were identified as DRACH motifs. DRACH were identified as being more specific, referring to motifs located in specific sequence contexts. Conversely, AC motifs were identified as encompassing all possible motifs.

### 2.6 Validation of Ψ

Ψ assessment and validation in mRNA were conducted using separate RNA extracts obtained from a different batch of plant material that had been submerged in sterile distilled water. The analysis was conducted using a modified sulfite/bisulfite treatment method - PRAISE method [67]. Briefly, 6 μl total RNA (550 ng) was dissolved in 50 μl of a mixture of freshly prepared potassium sulfite and sodium bisulfite (50:50, molar proportion) with the addition of 100 mM hydroquinone which is a 100:1 final mixture of bisulfite/sulfite solution and hydroquinone. After 5 h of incubation at 70°C, the mixture was desalted by passing it through Micro Biospin 6 chromatographic columns twice (Bio-Rad, 7326200). The desulfonation step was carried out by incubation of desalted RNA with an equal volume of 1.0 M Tris–HCl (pH 9.0) at 75 °C for 30 min. The reaction was then immediately stopped by chilling on ice and followed by RNA precipitation with 3 volumes of ethanol (99.9 %). Quantity of extracted RNA was measured with the Qubit™ RNA HS Assay Kit. Next, approximately 400 ng of total RNA was reverse transcribed into cDNA and amplified to prepare the cDNA library following the protocol specified by Oxford Nanopore Technologies (ONT) for cDNA-PCR Sequencing Kit (SQK-PCS114). Adapter-ligated cDNA sequences were sequenced on the MinION flow cell (vR10.4.1) with MinION Mk1C sequencing device.

### 2.7 Differential adenylation

The nanopolish v.0.14.1 [https://github.com/jts/nanopolish] was used to obtain information about the lengths of tails in individual transcripts. This analysis was performed by running the nanopolish index and nanopolish polya commands. The quality of nanopolish output files was analyzed using nanotail v.0.1.0 [https://github.com/smaegol/nanotail]. Only observations with qc_tag "PASS" and "SUFFCLIP" were qualified for further analysis. Modification sites with a probability greater than 0.6 were used for poly(A) tail analysis. The Wilcoxon test carried out in the R was used to determine the significance of changes in tail lengths between transcripts with identified RNA modifications and other non-modified transcripts of the same gene. A p-value < 0.05 was considered as significant. Additionally, a Cohen’s test was conducted where the effect size (r) was determined.

### 2.8 Transcript length, alternative splicing, G/C content

Transcript length, splicing event and G/C content were compared between transcripts with identified RNA modifications and their non-modified variants for the same genes. Transcript length analysis was performed based on genomic GTF annotations. For Ψ, transcripts were categorized as modified if at least one site had a probability greater than 0.8. In contrast, for m5C and m6A, transcripts were classified as modified when at least one position had a probability exceeding 0.6. The significance of differences in transcript lengths was assessed using the Wilcoxon test. A p-value of < 0.05 was considered statistically significant. Additionally, r was calculated for transcript length and GC ratio. Alternative splicing events were generated from the previously obtained GTF file using the SUPPA v.2.4 program [55]. The number of events in transcripts with RNA modifications was then compared to the number of events in other transcripts of the same gene. Splicing events were categorized into the following groups: Skipping Exon (SE), Mutually Exclusive Exons (MX), Alternative 5′ Splice Site (A5), Alternative 3′ Splice Site (A3), Retained Intron (RI), Alternative First Exon (AF), and Alternative Last Exon (AL). GC content was calculated in the R script based on the FASTA file of the transcriptome. The Wilcoxon test was conducted again, with a statistical difference defined as a p-value below 0.05. The proportions of modified nucleotides (Ψ, m6A, and m5C) to their respective unmodified bases (U, A, and C) in the transcript were calculated by dividing the number of detected modified nucleotides by the total number of the corresponding unmodified bases.

### 2.9 Functional annotations

All DEGs, transcripts with significant modification profile changes were annotated using BlastP v.2.12.0 [41]. Given the incomplete and uncharacterized status of many *M. polymorpha* gene symbols in databases, the identification of translated genes in *R. fluitans* was based on *A. thaliana* protein homology. A cut-off threshold of e-value < 10e-5 was set for blastp homology searching. The resulting gene signatures, including DEGs and other epitranscriptome candidates, were scanned for enrichment in Gene Ontology (GO) function annotations using the g:Profiler v.0.2.2 R library [30]. Biological processes (BP), cellular components (CC), and molecular functions (MF) were assigned as ontological terms to essential genes. Enrichment analysis with a false discovery rate (FDR) cut-off of < 0.05 was employed to identify significant GO and pathway annotations.

### 2.10 Polar metabolites

Polar metabolites were extracted from freeze-dried and pulverized thallus tissues (40 mg) using a methanol:water mixture (1:1, v/v) with ribitol (100 ug) as an internal standard at 70°C for 30 min. After centrifugation and chloroform extraction to remove non-polar compounds, the polar fraction was dried in a speed vacuum rotary evaporator. The metabolites were derivatized with *O*-methoxamine hydrochloride and *N*-methyl-*N*-trimethylsilyl-trifluoroacetamide, and the chromatographic analysis of the resulting derivatives was performed using a gas chromatograph GC-2010 with a quadrupole mass spectrometer (GCMS-QP2010 Plus, Shimadzu) equipped with a ZEBRON ZB-5MSi Guardian capillary column [15], [23], [51]. Metabolites were identified by comparing their mass spectra with the NIST 05 library. The significance of statistical differences in metabolites between aquatic and terrestrial *R. fluitans* in arbitrary units (a.u.) was calculated using the omu v.1.0.1 library, employing Welch's *t*-test and Bonferroni correction [54]. Differentially accumulated metabolites (DAMs) were classified as those metabolites with padj < 0.05.

### 2.11 Visualization

Visualizations were created in R environment v.4.3.1. The ggvenn v.0.1.10 library was used for Venn diagrams. ComplexHeatmap v.2.18.0 [17] was used to create heatmaps and upset plots. The rest of the visualizations were created using the ggplot2 v.3.5.1 package [56].

## 3 Results

### 3.1 Unveiling Ψ in *R. fluitans*: land vs. water adaptations

**Fig. 1:**
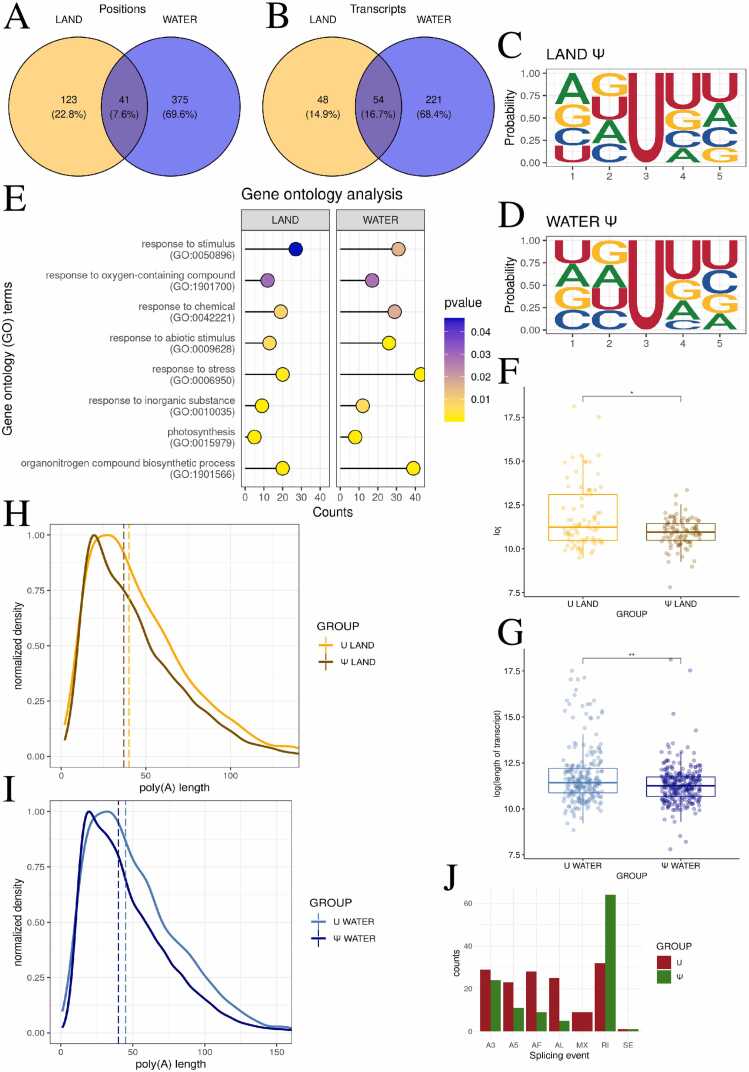
Ψ distribution and functional enrichment, with shorter transcript lengths in land and water *R. fluitans*. A,B - Venn diagram illustrating the number of pseudouridylated sites - A, transcripts - B in land (orange), water (blue), and both environments (darkblue). C,D - The logo diagram depicts the probability of a nucleotide appearing in the first five positions of the significant Ψ motifs in land (C) and water (D) environments. E – Lollipop chart illustrates the relationships between GO terms in the analysis, comparing land and water environments. The y-axis represents GO processes, while the x-axis shows the number of genes with detected pseudogenes involved in each process. Barplot colors are based on p-value significance. F,G - Boxplot charts depict the differences in log-transformed transcript length between Ψ-modified and non-Ψ-modified transcripts in two environments: land – F and water – G. H,I - Poly(A) tail profiling comparing transcripts with detected Ψ to other transcripts of the same gene in terrestrial (H) and aquatic (I) environments. J – Barplot of the distribution of splicing events in transcripts with Ψ (green color) vs. other transcripts of the same gene (red color).

Ψ modifications were detected in 311 and 824 transcripts in the land and water forms of *R. fluitans*, respectively. A total of 164 and 416 statistically significant Ψ sites were identified in the land and water forms of *R. fluitans*, respectively (Fig. 1A, Tabel S1,S2). Positions were assigned to transcripts, 102 transcripts were detected in terrestrial form and 275 in aquatic form, 54 transcripts were pseudouridine-enriched in both aquatic and terrestrial forms. (Fig. 1B, Table S1,S2). The transcripts exhibiting the highest levels of pseudouridylation in the aquatic form of *R. fluitans* were *CL.14105.3*, coded Fatty acid/sphingolipid desaturase and unannotated mRNA - *CL.24113.1*, both containing eight potential pseudouridylation sites. (Table S1) In the terrestrial form, other unannotated transcript *CL.3752.1* exhibited 19 potential Ψ sites, while *CL.14105.3* showed 9 (Table S2). A functional analysis was subsequently performed to gain a deeper understanding of the biological processes in which pseudouridylated transcripts are involved. It was hypothesized that such information would be crucial to elucidating the role of this process in *R. fluitans*. 5-nucleotide motifs were analyzed, where Ψ was always present in the 3rd (middle) position. In both the aquatic and terrestrial *R. fluitans*, guanine was most frequently found at the 2nd position, while uridine was most often observed at the 4th and 5th positions within the motif. In the terrestrial *Riccia*, the first position in the motifs was most frequently occupied by adenine, whereas in the aquatic *Riccia*, it was uracil (Fig. 1C, 1D). Genes from the terrestrial form (65 genes) and the aquatic form (191 genes) of *R. fluitans* were included in the GO analysis. In the land form 193 significant processes were identified and classified into 48 biological processes (BP), 84 cellular components (CC), and 61 molecular functions (MF). In the water form of *R. fluitans*, 360 significant processes were detected, comprising 161 BP, 98 CC, and 101 MF. Among the statistically significant processes identified for both groups were photosynthesis (GO:0015979), response to stress (GO:0006950), and response to stimulus (GO:0050896) (Fig. 1E, Table S3,S4).

The influence of Ψ on transcript length was subsequently investigated. Transcripts harbouring Ψ were observed to be shorter than transcripts of the same gene lacking a Ψ site. The difference in transcript lengths was found to be statistically significant for both the land (p = 0.0156, r = 0.179, small effect) and the water forms (p = 0.00323, r = 0.130, small effect) (Fig. 1F,1G, Table S5,S6). Since the analysis focused on splice transcripts, it did not consider poly(A) tails, so we aimed to investigate how the length of this dynamic structure changes. The length of the poly(A) tail and its correlation with transcripts containing Ψ were examined. The length of tails was found to be statistically significantly different between Ψ-containing transcripts and other transcripts of the same gene for both land (p < 0.001, r = 0.0372, small effect) and water (p < 0.001, r = 0.0468, small effect) (Figs. 1H,1I, Table S7,S8). In both cases, a shortening of tails was observed in Ψ-containing transcripts. It was deemed reasonable to associate transcript length with the occurrence of alternative splicing. Alternative splicing, which can influence transcript length, was investigated. Analysis of pseudouridylated transcripts revealed Ψ sites in transcripts with splicing events. No MX events were detected in these transcripts, while 9 were identified in the unmodified transcripts. A twofold increase in RI was observed in the Ψ transcripts (64) compared to the unmodified ones (32). The number of A3 events was similar in both groups, with 24 in the Ψ transcripts and 29 in the unmodified transcripts. Additionally, only one SE was detected in both groups. Events A5 (23), AF (28), and AL (25) were more frequent in the unmodified transcripts, with respective frequencies of 11, 9, and 5 in the Ψ transcripts (Fig. 1J).

The distribution of nucleotides in modified transcripts was also under consideration. It was assumed that the G/C content and the ratio of modified to unmodified nucleotides within the transcript would be sufficient to assess whether modified transcripts were more prone to modification due to a higher frequency of the nucleotide undergoing modification. A statistically significant difference in G/C content was found between Ψ-containing transcripts and other transcripts of the same gene, in both terrestrial (p < 0.001, r = 0.315, moderate effect) and aquatic (p < 0.001, r = 0.252, small effect) forms. The pseudouridine-burdened transcripts were found to have a lower G/C content, which actually means that after nucleotide distribution, they had a greater chance of pseudouridinylation. The percentage of Ψ relative to all uridines in the transcripts was determined to be 0.001 % in the land form and 0.002 % in the water form (Fig. S1A,S1B, Table S10,S11).

To confirm the reliability of the high throughput direct RNA sequencing results, additional validation of RNA modification was performed. The localization of Ψ on transcripts was validated using the PRAISE method. This method allows for the detection of Ψ by observing deletions at specific positions. A total of 42 positions in the aquatic form of *R. fluitans*, where Ψ had previously been detected through direct RNA sequencing, were confirmed (Fig. S2).

### 3.2 The impact of m5C methylation on transcript elongation in *R. fluitans*

**Fig. 2:**
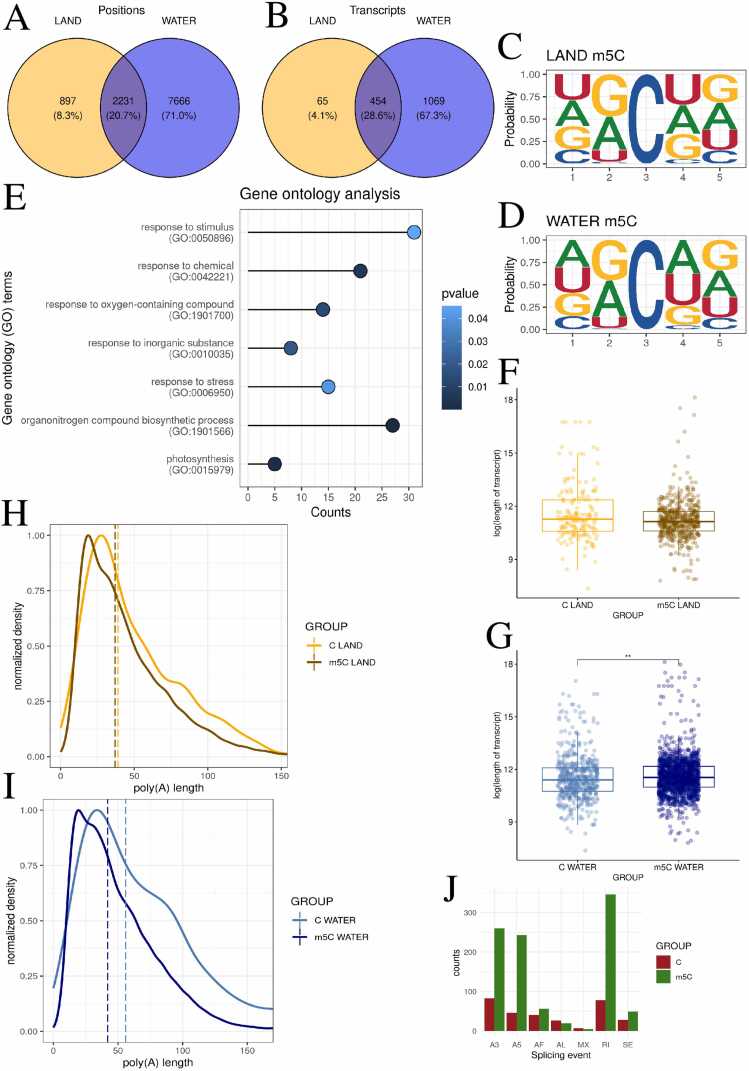
m5C methylation patterns and differential transcript elongation in land and water *R. fluitans.* A,B - Venn diagram illustrating the number of methylated sites - A, transcripts - B in land (orange), water (blue), and both environments (darkblue). C,D - The logo diagram depicts the probability of a nucleotide appearing in the first five positions of the significant m5C motifs in land (C) and water (D) environments. E – Lollipop chart shows selected statistically significant GO processes on the y-axis, while the x-axis represents the number of m5C-modified genes involved in each process. p-values are represented by a blue scale, where dark blue indicates lower p-values. F,G - Boxplot charts depict the differences in log-transformed transcript length between m5C-modified and non-m5C-modified transcripts in two environments: land – F and water – G. H,I - Poly(A) tail profiling comparing transcripts with detected m5C to other transcripts of the same gene in terrestrial (H) and aquatic (I) environments. J – Barplot of the distribution of splicing events in transcripts with m5C (green color) vs. other transcripts of the same gene (red color).

AC analysis of m5C methylation revealed 26,330 putative methylation sites in *R. fluitans* habited in terrestrial environments and 85,295 growing in aquatic conditions. A total of 3128 sites with a probability of greater than 0.6 were identified in land and 9897 in water environments (Fig. 2 A,2B, Table S12,S13). The 5-nucleotide motifs with m5C were also examined, where the modified cytosine was always present in the central (third) position. Guanine in the second and fifth positions was the most frequent nucleotide in both environments. In the first and fourth positions, uracil was the most common nucleotide in terrestrial *Riccia*, while adenine was most frequent in the aquatic *Riccia* (Fig. 2 C,2D). To comprehensively analyze and compare the impact of the modification, similar analyses were conducted for m5C as for Ψ. The analysis of transcript lengths with detected m5C, and other transcripts from the same gene, provided information about the lack of significant differences (p = 0.0537, r = 0.0731, small effect) in transcript length in the terrestrial environment (Fig. 2 F, Table S14). However, significant increase in the length of m5C-containing transcripts was observed in the aquatic environment compared to other transcripts from the same genes (p = 0.009, r = 0.0573, small effect) (Fig. 2 G, Table S15). It was decided again to check whether there were significant differences in poly(A) tail lengths. The analysis of poly(A) tail lengths revealed a shortening of the poly(A) tails in transcripts modified with m5C (aquatic: p < 0.001, r = 0.0379, small effect, terrestrial: p = 0.021, r = 0.0164, small effect) (Figs. 2H, 2I, Table S16,S17). Due to the increasing length of aquatic transcripts modified with m5C, it was decided to investigate the impact of this modification on alternative splicing. A higher number of RI events was detected in m5C-modified transcripts (346) versus other transcripts (78). A similar number of MX events was detected (5 for m5C-modified transcripts and 7 for other transcripts), as well as AL events (19 for m5C-modified transcripts and 27 for other transcripts) and SE events (49 for m5C-modified transcripts and 28 for other transcripts). Other splicing events detected by the SUPPA program, such as A3, A5, and AF, showed much higher frequencies in m5C-modified transcripts: A3 - 260, A5 - 243, AF - 56, compared to A3 - 83, A5 - 46, and AF - 40 in transcripts without this modification (Fig. 2 J, Table S18).

To examine the nucleotide composition in transcripts, analyses of G/C content and the ratio of modified cytosines to unmodified cytosines within the transcriptome were conducted once more. Transcripts with detected m5C modification were found to be significantly different in G/C content from other transcripts of the same gene in both land (p < 0.001, r = 0.259, small effect) and water (p < 0.001, r = 0.257, small effect). Lower G/C content was observed in transcripts with detected modification. Transcripts with m5C were statistically less likely to have a methyl group added at the modification site. The proportion of m5C to total cytosines in the transcript was determined to be 0.08 % under aquatic conditions and 0.03 % under terrestrial conditions (Fig. S1C,S1D, Table S19,S20).

Next, the CHEUI software was employed to perform a differential analysis between the positions detected in both groups. It was decided to utilize this option to observe in detail how specific positions were changed under the influence of stoichiometry (the percentage of modified reads at the site). The 21 532 common sites were qualified for differential analysis. 394 sites were qualified as statistically significant, of which 164 were classified as lower and 230 as upper (Fig. S3, Table S21). The positions were assigned to 92 genes, which were used for GO analysis, revealing significant processes: response to stimulus (GO:0050896), response to stress (GO:0006950), response to oxygen-containing compounds (GO:1901700), and response to chemicals (GO:0042221) (Fig. 2E, Table S22).

### 3.3 Analysis of m6A methylation motifs reveals distinct patterns in land and aquatic environments

**Fig. 3:**
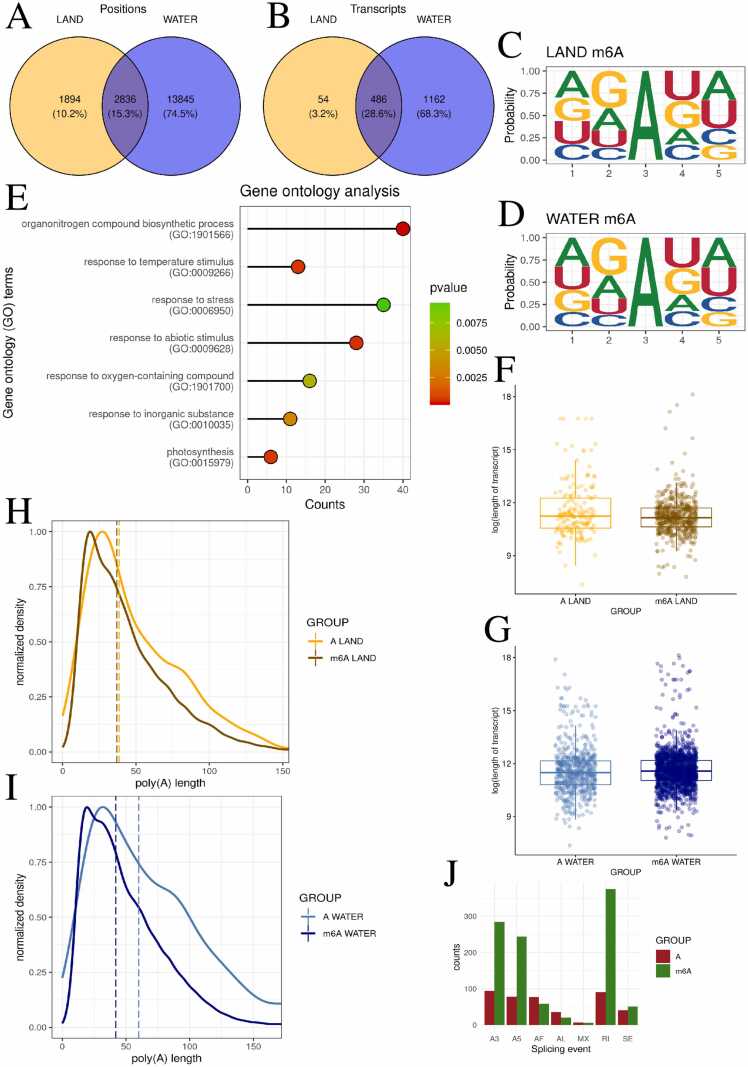
Distinct m6A methylation motifs and shared transcripts revealed in land and aquatic *R. fluitans.* A,B - Venn diagram illustrating the number of methylated sites - A, transcripts - B in land (orange), water (blue), and both environments (darkblue). C,D - The logo diagram depicts the probability of a nucleotide appearing in the first five positions of the significant m6A motifs in land (C) and water (D) environments. E – Lollipop chart shows selected statistically significant GO processes on the y-axis, while the x-axis represents the number of m6A-modified genes involved in each process. The p-values are represented by a blue scale, where dark blue indicates lower p-values. F,G - Boxplot charts depict the differences in log-transformed transcript length between m6A-modified and non-m6A-modified transcripts in two environments: land – F and water – G. H,I - Poly(A) tail profiling comparing transcripts with detected m6A to other transcripts of the same gene in terrestrial (H) and aquatic (I) environments. J – Barplot of the distribution of splicing events in transcripts with m6A (green color) vs. other transcripts of the same gene (red color).

A comprehensive analysis of m6A methylation across plants occupying different environments uncovered 33,175 land-based and 112,823 aquatic methylation sites. The analysis of AC motifs conducted with the CHEUI program revealed 16,681 statistically significant positions in the aquatic habitats of *R. fluitans*, while 4730 significant positions were identified in terrestrial environments (Fig. 3A, Table S23,S24). The analysis revealed the presence of these positions across 540 transcripts in the terrestrial form and 1648 transcripts in the aquatic form of *R. fluitans* (Fig. 3B). In contrast to Ψ and m5C, the most frequently occurring nucleotides in the 5-nucleotide motif, where the methylated adenine is always in the middle (3rd position), were found to be most similar between water and land. The most frequently occurring nucleotide in the first position was adenine, in the second position guanine, in the fourth position uridine, and in the fifth position adenine, both in the aquatic *Riccia* and the terrestrial *Riccia*. (Figs. 3C, 3D) Transcript lengths were compared between transcripts harboring m6A modifications and those without m6A modifications from the same genes. No significant differences in transcript lengths were revealed when comparing transcripts harboring m6A versus non-m6A transcripts from the same genes (p = 0.15, r = 0.0536, small effect, for land, p = 0.06, r = 0.0386, small effect for water) (Figs. 3F, 3G, Table S25,S26). Similarly to Ψ and m5C, the tail lengths were found to be statistically shorter in transcripts affected by the m6A modification (p = 0.0484, r = 0.0140, small effect for land, p < 0.001, r = 0.0404, small effect for water) (Figs. 3H, 3I, Table S27, S28). Alternative splicing was examined in transcripts with and without the m6A modification in the same genes. A significant increase in RI events was observed (375 vs. 90 in other transcripts), A3 events (284 vs. 94 in other transcripts), and A5 events (244 vs. 78 in other transcripts). A similar number of splicing events was recorded for MX (6 for m6A transcripts, 7 for non-m6A transcripts), SE (51 for m6A transcripts, 40 for non-m6A transcripts), AF (58 for m6A transcripts, 77 for non-m6A transcripts), AL (20 for m6A transcripts, 35 for non-m6A transcripts). (Fig. 3J, Table S29).

A comprehensive analysis of nucleotide distribution revealed that the GC content was downgraded for transcripts with m6A modifications in both terrestrial (p < 0.001, r = 0.264, small effect) and aquatic (p < 0.001, r = 0.243, small effect) environments. The percentage of m6A relative to all adenines in the all transcript was determined to be 0.04 % under terrestrial conditions and 0.1 % under aquatic conditions (Fig. S1E,S1F, Table S30,S31). A differential methylation analysis targeting 26,981 sites identified in both groups revealed that 612 of these sites exhibited significant differences in m6A methylation. Of these, 247 m6A positions were found to display lower stoichiometry, while 365 sites indicated higher stoichiometry (Fig. S4, Table S32). These significant sites were associated with 195 genes, which were found to be functionally enriched in processes related to response to abiotic stimulus (GO:0009628), response to stress (GO:0006950), response to oxygen-containing compound (GO:1901700), and photosynthesis (GO:0015979) (Fig. 3E, Table S33).

Motif enrichment analysis utilizing DRACH and AC motifs identified 4 and 39 transcripts, respectively, that were common between the terrestrial and aquatic forms of *R. fluitans*. Furthermore, 156 statistically significant m6A RNA modification sites were identified in the aquatic *R. fluitans* species by the NanoSPA program, and 173 such sites were identified by the m6Anet software. In the terrestrial *R. fluitans* ecotype, the 68 and 27 statistically significant m6A modification sites were identified by both programs, respectively (Fig. S5).

### 3.4 Analysis of gene expression differences in *R. fluitans* under aquatic and terrestrial conditions

**Fig. 4:**
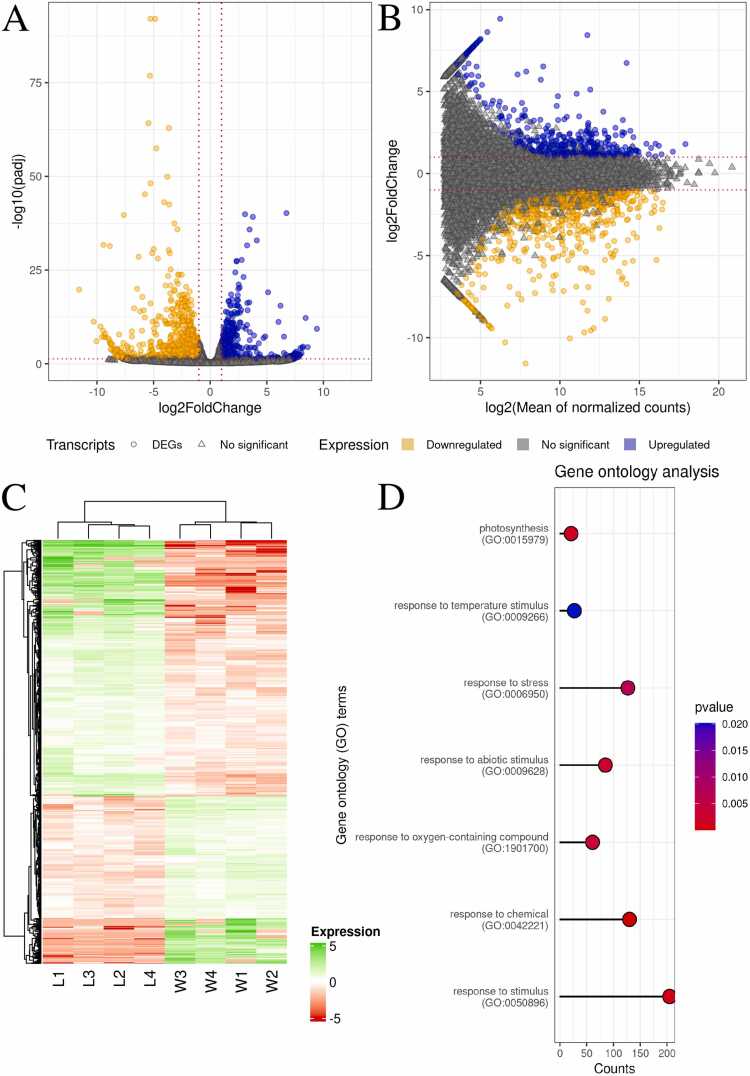
Differential gene expression between terrestrial and aquatic *R. fluitans*. A - Volcano plot showing log2 fold change (x-axis) and -log10(q-value) (y-axis). B - MA plot showing log2 mean of normalized counts (x-axis) and log2 fold change (y-axis). Blue circles represent upregulated genes, and orange circles represent downregulated genes. Gray triangles indicate genes with statistically insignificant changes. C - Heatmap showing the expression of 1226 differentially expressed genes. Darker shades of green indicate higher z-scored expression, and shades of red indicate lower expression. D - Lollipop chart representing 7 selected GO processes (y-axis) and the number of differentially expressed genes involved in them (x-axis). The lollipop color corresponds to the p-value, according to the blue-to-red scale shown on the right.

Additionally, part of our analyses was the analysis of gene expression in *R. fluitans*. The analysis focused on differences in expression between the aquatic and terrestrial forms of *R. fluitans*. A total of 1226 DEGs were detected, of which 741 were downregulated and 485 were upregulated (Figs. 4A, 4B, 4C, Table S34). The highest expressing DEGs in aquatic *Riccia* were *CL.14105* (fatty acid/sphingolipid desaturase) and *CL.11766* (fructose-1,6-bisphosphate aldolase 2), while in terrestrial *Riccia*, the most highly expressed DEGs that were annotated in *A. thaliana* were *CL.21420* (pyridoxal phosphate (PLP)-dependent transferases superfamily protein) and *CL.12906* (plasma membrane intrinsic protein 1B). 612 DEGs were annotated with data from *A. thaliana*, and these molecules were involved in 623 GO processes, including response to stimulus (GO:0050896), response to chemical (GO:0042221), and response to stress (GO:0006950) (Fig. 4D, Table S35).

### 3.5 Integrated analysis of epitranscriptomic modifications and gene expression

**Fig. 5:**
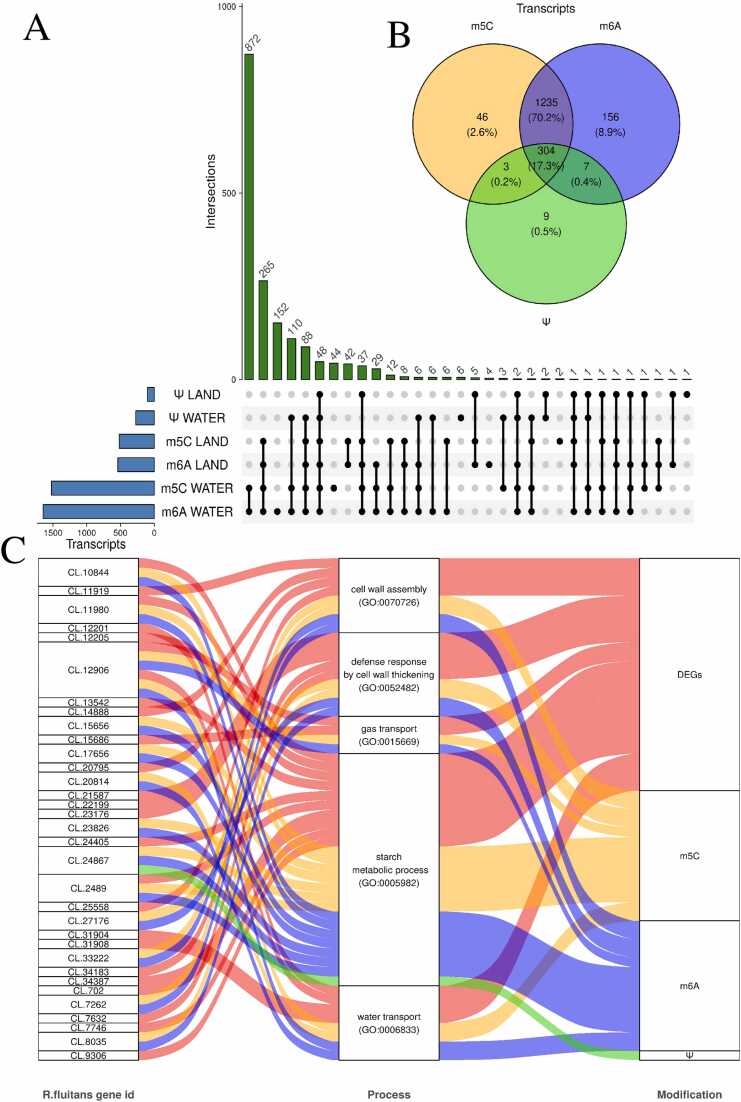
Analysis of RNA Modifications and Their Associations with Genes and Biological Processes. A - The upset plot displays the transcripts shared across all modifications. The blue bar plots represent the number of transcripts for each modification, while the green ones indicate those that are jointly compatible, marked by filled black dots. B - The Venn diagram illustrates the relationships between the occurrence of modifications in transcripts for m5C (orange), m6A (blue), and Ψ (green). C - The Sankey diagram illustrates the relationships between the identified GO terms, DEGs (red), transcripts with Ψ (green), m5C (orange), and m6A (blue) modifications. The "*R.fluitans* gene id" column provides gene identifiers. The "Process" column corresponds to the selected 5 GO processes. The "Modification" column represents genes with a specific RNA modification or that are DEGs.

A comprehensive analysis of shared epitranscriptome features was conducted using multiple software tools and methylation detection techniques. The results revealed that m5C and m6A modifications exhibited the highest degree of overlap, likely due to the predominance of the AC method. The DRACH motif was not the primary focus in the detection of modifications (Figs. 1C, 1D, 2C, 2D, 3C, 3D). The aquatic condition induced the highest number of m6A and m5C modifications within 872 unique transcripts modified in *R. fluitans*. Other 265 unique transcripts were modified by methylation of m5C and m6A in both environments. All three RNA modifications (Ψ, m6A, and m5C) were detected in 48 unique transcripts across both habitats of *Riccia* (Fig. 5A). In total, 304 transcripts were found to contain all three investigated modifications. Additionally, a smaller number of unique transcripts were identified between Ψ and m5C (3, 0.2 %) and Ψ and m6A (7, 0.4 %). A total of 1235 (70.2 %) common transcripts were detected between the transcripts modified by m5C and m6A. Transcripts modified by only one modification were detected as follows: 46 (2.6 %) by m5C, 156 (8.9 %) by m6A, and 9 (0.5 %) by Ψ (Fig. 5B).

It was revealed by the analysis of gene identification with RNA modifications and differentially expressed genes (DEGs) that 997 genes exhibited RNA modifications, and 1226 genes showed significant differences in expression levels. Variability at different levels, both in RNA modifications and gene expression, was allowed for examination by this analysis. Surprisingly, changes in both expression and RNA modifications were shown by only 121 genes (constituting a small fraction of the total). It is suggested by this finding that RNA modifications and changes in gene expression may occur independently of each other, with only a small overlap. A comprehensive analysis and a better understanding of the complex mechanisms of gene regulation are allowed for by this approach (Fig. S6).

Results from the analyses were combined to scan the relevant GO processes associated with the growth of *R. fluitans* in both aquatic and terrestrial environments. The following processes were selected: cell wall assembly (GO:0070726), defense response by cell wall thickening (GO:0052482), gas transport (GO:0015669), starch metabolic process (GO:0005982), water transport (GO:0006833). The analysis showed that changes in gene expression (DEGs) as well as post-transcriptional RNA modifications (m5C, m6A, Ψ) occur in the studied biological processes. The most modification of m5C and m6A were observed in the starch metabolic process, suggesting that these modifications may play a significant role in regulating this process. Additionally, DEGs were identified in each of the processes, indicating a potential impact of these changes on biological functions (Fig. 5C, Table S36, S37).

### 3.6 The impact of environmental conditions on metabolism in *R. fluitans*

**Fig. 6:**
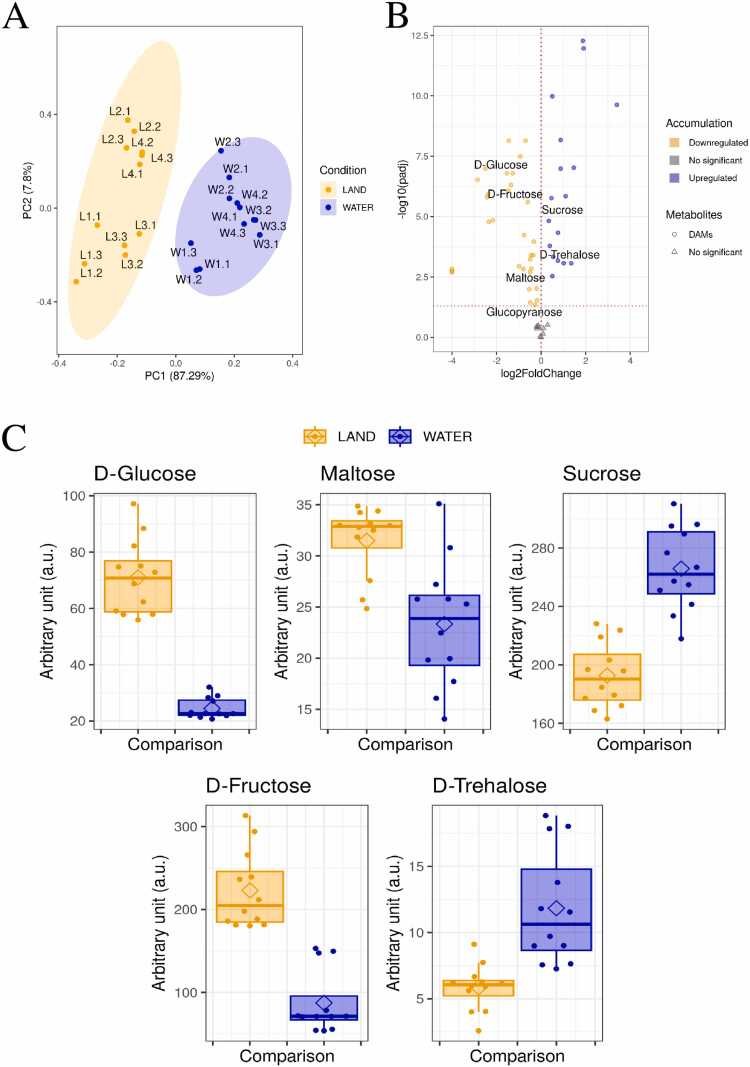
Metabolic profiling of terrestrial and aquatic *R. fluitans*. A - PCA plot showing the reduction of data to two dimensions: PC1 (x-axis) and PC2 (y-axis). Orange points represent terrestrial *R. fluitans*, and blue points represent aquatic *R. fluitans*. B - Volcano plot showing the relationship between padj (y-axis) and log2FoldChange (x-axis). Blue points indicate metabolites with increased accumulation in aquatic *R. fluitans* (upregulated DAMs), and orange points indicate metabolites with increased accumulation in terrestrial *R. fluitans* (downregulated DAMs). C - Boxplots showing differences in Arbitrary units between terrestrial (orange) and aquatic (blue) *R. fluitans* for D-Glucose, Maltose, Sucrose, D-Fructose, and D-Trehalose.

Chromatographic analysis enabled the detection of 57 polar metabolites accumulated in both aquatic and terrestrial environments, of which 37 were identified (Table S38). Principal component analysis (PCA) performed on all detected metabolites revealed a clear differentiation between the aquatic and terrestrial *R. fluitans* groups (Fig. 6A). Furthermore, significant differences were observed in 48 of these metabolites between the two environments. Among the metabolites that showed statistically significant differences, the largest group consisted of soluble carbohydrates. Previous analyses had demonstrated significant differences in the expression and RNA modifications of genes involved in starch metabolism, leading to a detailed examination of sugars directly or closely implicated in this pathway, specifically maltose, D-glucose, sucrose, D-trehalose, and D-fructose. Significant differences between the aquatic and terrestrial environments were observed for all these sugars (Figs. 6B, 6C). Specifically, D-glucose, maltose, and D-fructose exhibited significantly higher levels in the terrestrial form of *R. fluitans*, whereas sucrose and D-trehalose showed higher levels in the aquatic form (Fig. 6C).

## 4 Discussion

Epitranscriptomic changes in *Riccia fluitans* clearly demonstrate that RNA modifications are dynamically regulated in response to environmental conditions and may constitute an important layer of gene expression control enabling phenotypic plasticity. Our results indicate that the aquatic form of *R. fluitans* exhibits a substantially elevated levels of three major RNA modifications—N6-methyladenosine (m6A), 5-methylcytosine (m5C), and pseudouridine (Ψ)—compared to its terrestrial counterpart. This enrichment suggests that the aquatic environment induces epitranscriptomic reprogramming as part of a broader adaptation strategy to prolonged submergence and associated stresses. Detection of RNA base modifications using DRS is rapidly advancing, particularly for adenine methylation [1], [22], [26]. However, identification models for Ψ and m5C using the R9.4.1 pore are limited [27]. These modifications can be validated through third-generation sequencing employing an adapted PRAISE method, which identifies Ψ positions via characteristic deletions at modification sites and m5C via C-to-T conversion ratios. Deletions at Ψ positions in *R. fluitans* transcripts from an aquatic environment were identified using the PRAISE method, thereby confirming the presence of Ψ at these sites. This method also validated m5C sites detected by DRS. These results support the application of third-generation sequencing in conjunction with the PRAISE method for accurate mapping of pseudouridylation and m5C sites [67] using high-throughput and more accurate R10.4.1 flow cells. Recent improvements in the Nanopore Dorado basecaller paired with the latest RNA004 chemistry have enabled detection of a wide range of RNA modifications—including m6A, m5C, inosine, and Ψ—across all sequence contexts with over 99 % confidence [https://github.com/nanoporetech/dorado].

RNA modifications are increasingly recognized as important modulators of stress responses in plants. Studies in other species, including *Hippophae rhamnoides* [66], *Populus* [69], and *Nicotiana tabacum* [59], have shown that levels of m6A can increase or decrease depending on the type of stress and plant species, indicating that regulation of RNA methylation is context-dependent. In our study, m6A levels were significantly elevated in the aquatic form of *R. fluitans*, which is consistent with observations in tobacco under salt stress [59] but differs from reports in drought-stressed trees where a decrease was observed [66], [69]. This supports the notion that the direction of m6A modulation may reflect distinct physiological needs: in aquatic environments, enhanced m6A levels could facilitate rapid transcript turnover or fine-tuning of stress-responsive gene expression. Similarly, m5C modifications were more abundant in the aquatic form, reaching a level of 0.08 % of total cytosines, nearly three times higher than in the terrestrial form (0.03 %). These values are comparable or even slightly higher than those reported for *Arabidopsis thaliana* using nanopore-based methods [10], suggesting that m5C plays a functionally relevant role in early land plants as well. Functional enrichment of m5C-modified transcripts in our study indicates a strong association with stress-related processes (e.g., GO:0006950 – "response to stress" and GO:1901700 – "response to oxygen-containing compounds"). These findings align with previous evidence that RNA methylation may help regulate oxidative stress and redox homeostasis [10]. Ψ modification, although the rarest of the three analyzed, was also significantly more abundant in the aquatic form. This modification has been shown to influence RNA structure and stability and is enriched in mRNAs related to photosynthesis and stress responses in *Arabidopsis* [50]. Our data are consistent with these findings: Ψ-marked transcripts in *R. fluitans* were functionally enriched in categories associated with plastid activity, cell wall processes, and response to water deficit, indicating a possible contribution to flooding tolerance.

In addition to epitranscriptomic changes, we observed profound metabolic and transcriptional differences between the terrestrial and aquatic forms, especially in carbohydrate metabolism. The terrestrial form accumulated more simple sugars such as glucose, fructose, and maltose, likely reflecting starch degradation and osmoprotective responses to water deficit. In contrast, the aquatic form had higher levels of sucrose and trehalose, consistent with findings in other species subjected to flooding or salinity stress [20], [45], [63]. Sucrose is not only an energy source but also a signaling molecule involved in regulating photosynthesis and stress responses. Trehalose, though present in low concentrations, has a well-documented role in stress protection and ROS scavenging [14], [60]. Importantly, these metabolic shifts are paralleled by changes in gene expression and RNA modification of key enzymes. For example, the gene encoding the large subunit of ADP-glucose pyrophosphorylase (AGPase; CL.24867), a central enzyme in starch biosynthesis, showed upregulation and increased m5C and m6A methylation in the aquatic form. AGPase is known to be regulated in response to environmental stimuli [32], and its epitranscriptomic modulation may allow rapid adjustment of carbohydrate metabolism under submerged conditions. Another interesting case is the UDP-glycosyltransferase gene CL.11980, which was both differentially expressed and modified in aquatic plants. UGTs play essential roles in the detoxification of xenobiotics, the glycosylation of secondary metabolites, and the modulation of hormone activity [46], [65]. Upregulation and modification of this gene may indicate enhanced need for glycosylation under aquatic conditions, possibly to stabilize or store stress-related metabolites. Adaptation to aquatic life also involves substantial changes in the cell wall, which serves as a key interface with the external environment. Our results indicate that aquatic plants display upregulation and RNA modification of genes involved in cell wall organization, such as those associated with GO:0052482 ("cell wall thickening") and GO:0070726 ("cell wall organization or biogenesis"). Among these were ABC-2 type transporter genes (CL.27176, CL.8035), both members of the pleiotropic drug resistance (PDR) family. These proteins are known to be involved in transport of secondary metabolites and may contribute to reinforcement of the cell wall or the secretion of defensive compounds under stress [19], [9]. Additionally, several receptor-like kinases with leucine-rich repeats (LRR-RLKs) were both differentially expressed and RNA-modified. These kinases are involved in sensing environmental cues and transducing stress signals, and they also play roles in regulating growth, immunity, and cell wall remodeling [35], [58]. Their modification and upregulation in the aquatic form suggest that they may mediate signaling pathways critical for adaptation to submergence.

Taken together, our findings reveal that *R. fluitans* undergoes profound molecular and metabolic reprogramming when transitioning between terrestrial and aquatic habitats. This includes changes in transcript abundance, carbohydrate metabolism, and post-transcriptional regulation via RNA modifications. The strong environmental dependence of m6A, m5C, and Ψ levels and their specific association with genes involved in key adaptive processes underscore the likely functional relevance of these epitranscriptomic marks. These results provide new insights into how early land plants may have developed mechanisms of phenotypic plasticity through epitranscriptomic regulation. Integration of RNA modification and differential gene expression data indicated limited overlap, implying that post-transcriptional and transcriptional regulatory layers may function largely independently. Moreover, they suggest that RNA modifications, rather than serving merely structural or stabilizing roles, may be central to dynamic and reversible responses to environmental change. Future studies combining functional analysis of specific RNA methylation events with phenotypic assays will be essential to elucidate the causal roles of individual modifications in stress adaptation.

## CRediT authorship contribution statement

**Mateusz Maździarz:** Writing – review & editing, Writing – original draft, Visualization, Validation, Supervision, Software, Resources, Methodology, Investigation, Formal analysis, Data curation, Conceptualization. **Katarzyna Krawczyk:** Writing – review & editing, Writing – original draft, Resources, Methodology. **Joanna Szablińska-Piernik:** Writing – original draft, Methodology, Investigation. **Łukasz Paukszto:** Writing – original draft, Software, Methodology. **Monika Szczecińska:** Writing – original draft, Methodology. **Paweł Sulima:** Writing – original draft, Methodology, Investigation. **Jakub Sawicki:** Writing – review & editing, Writing – original draft, Software, Project administration, Methodology, Funding acquisition, Conceptualization

## Declaration of Competing Interest

The authors state there is no conflict of interest.

## Data Availability

The raw reads were deposited in the ENA EMBL-EBI database at the following numbers PRJEB72691, PRJEB72692 and PRJEB97949. The genome and gtf file were deposited in the figshare database at the following link: https://figshare.com/projects/Riccia_fluitans/201057.

## Footnotes

Appendix A Supplementary data associated with this article can be found in the online version at doi:10.1016/j.csbj.2025.10.002.
