# Supplementary material for: Epitranscriptomic and expression profiling in Riccia fluitans across diverse environmental conditions
Source: Comput Struct Biotechnol J. 2025 Oct 2;27:4232–45. doi: 10.1016/j.csbj.2025.10.002 (PMC12513226; doi:10.1016/j.csbj.2025.10.002)
Supplement: Supplementary file 1 — Supplementary material [file mmc1.docx]

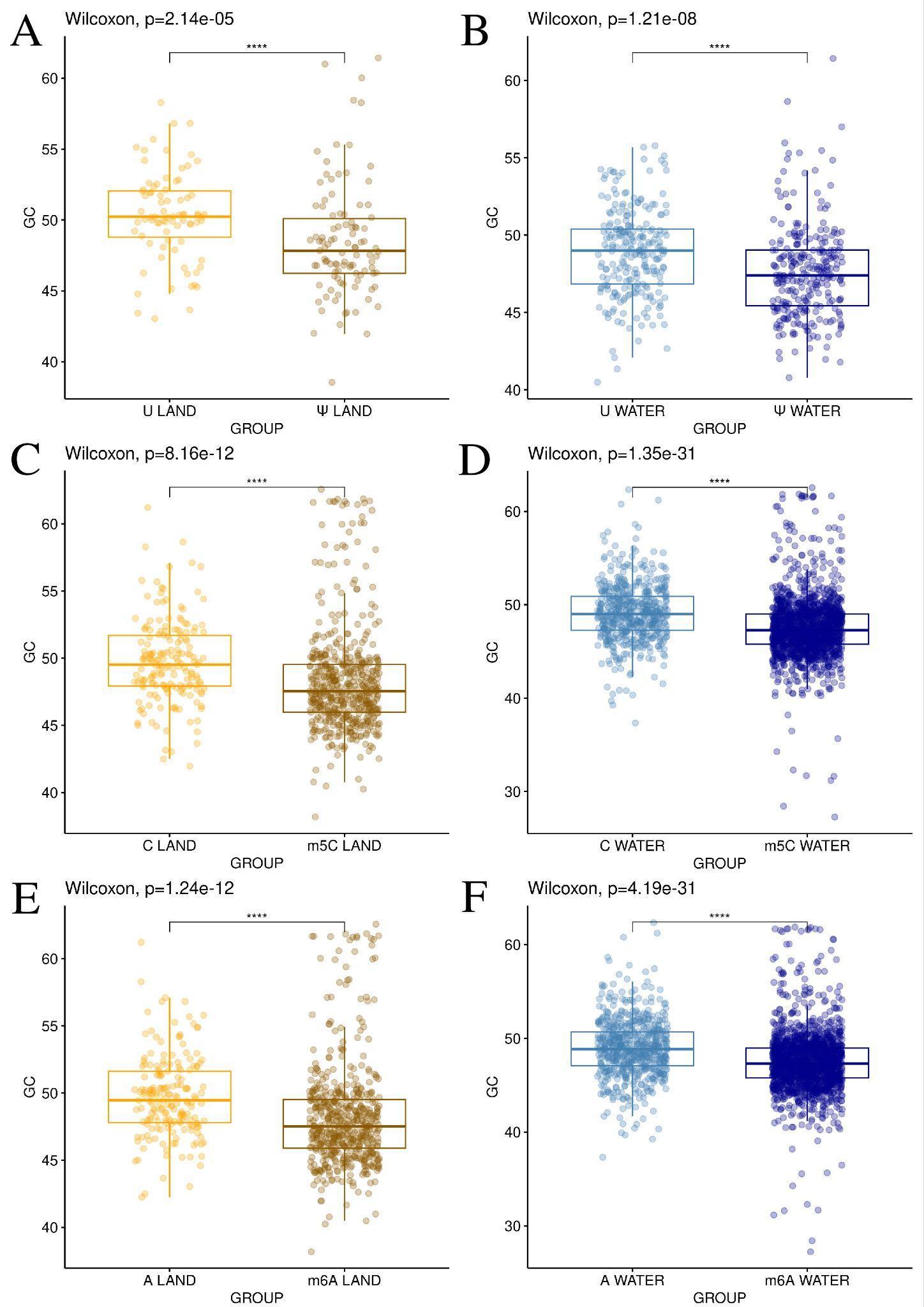


Figure S1. G/C content variation in RNA transcripts with different modifications in *R. fluitans* under diverse environmental conditions.

Boxplots reveal differences in G/C content between RNA A,B-modified transcripts for Ψ, C,D-modified transcripts for m5C, E,F-modified transcripts for m6A, and other transcripts of the same gene in different environmental conditions A,C,E for land and B,D,F for water *R. fluitans*. G/C ratio is on the y-axis, and transcript groups are separated on the x-axis.


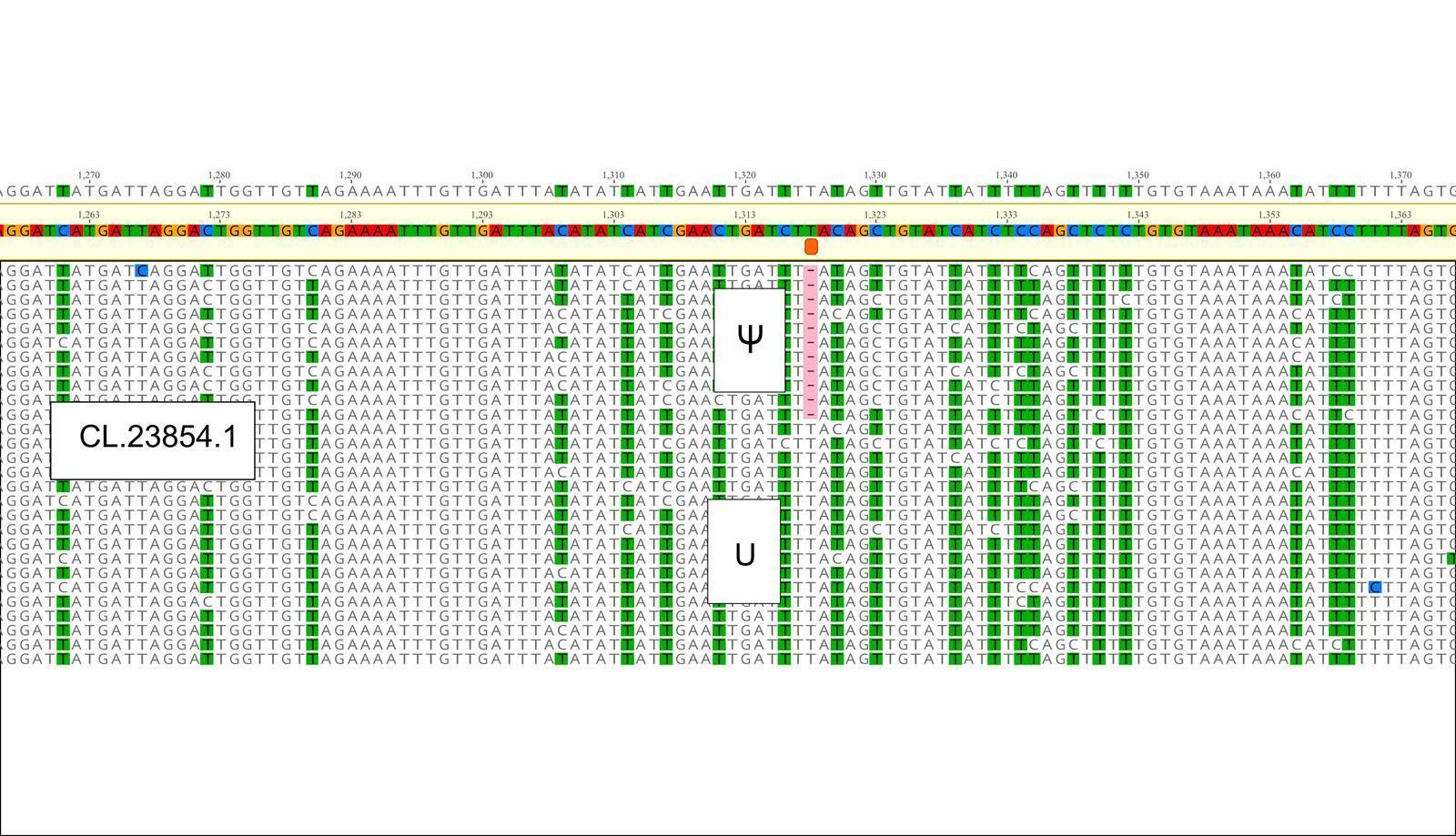


Figure S2. Visualization of CL.23854.1 transcript sequenced using PRAISE method adapted to nanopore sequencing technology. Presence of Ψ resulted in deletion at position 1318 which is congruent with NanoSPA prediction (probability 0.875).


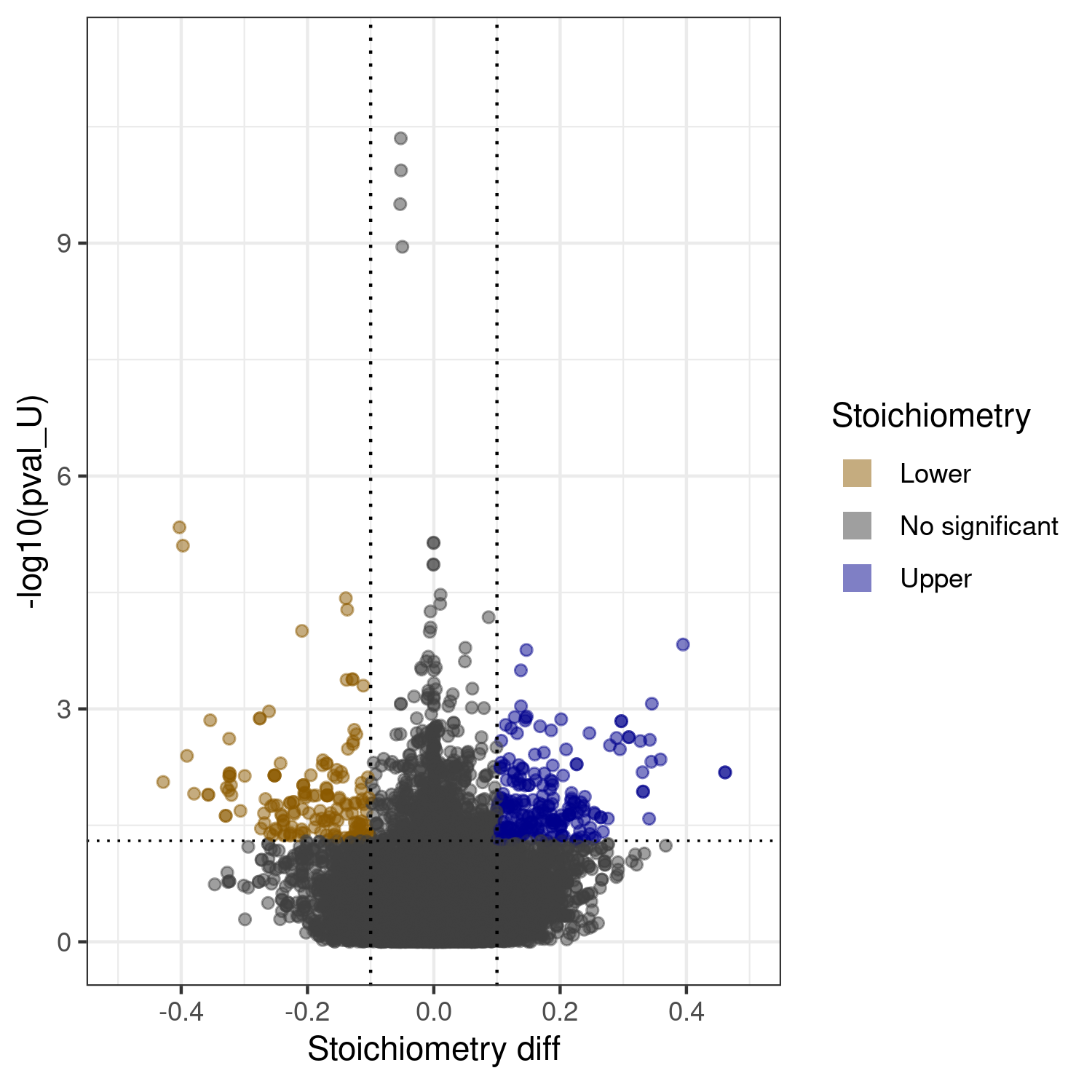


Figure S3. Volcano plot displays the methylation difference (stoichiometry diff) for each m5C site. The x-axis represents the magnitude of these differences, while the y-axis shows the negative log-transformed pval_U. Sites with statistically significant methylation changes are highlighted by orange (lower) and blue (upper) color. The horizontal dashed line indicates the adjusted p-value cutoff (0.05). The vertical dashed lines represent a stoichiometry diff threshold (abs(0.1)).


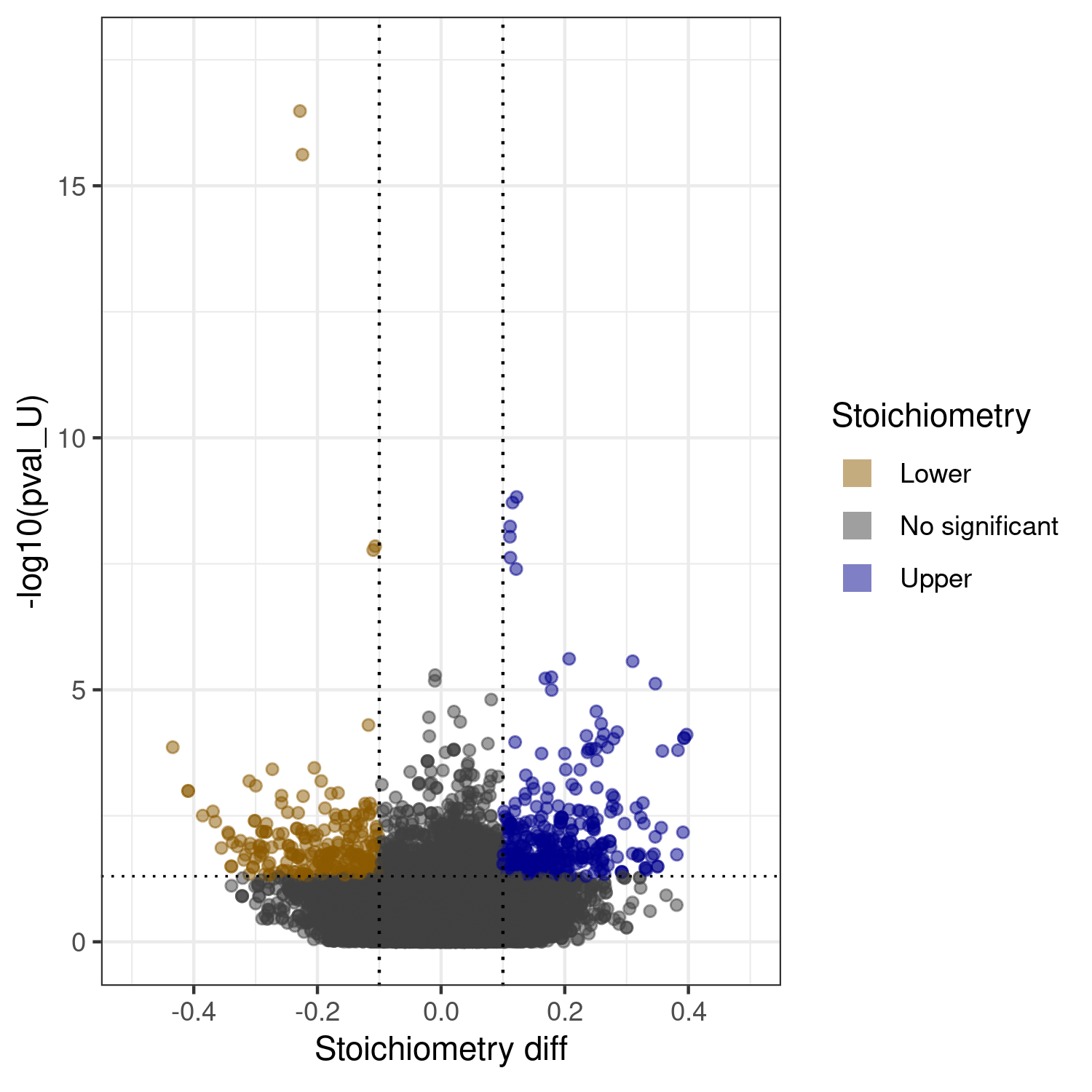


Figure S4. Volcano plot displays the methylation difference (stoichiometry diff) for each m6A site. The x-axis represents the magnitude of these differences, while the y-axis shows the negative log-transformed pval_U. Sites with statistically significant methylation changes are highlighted by orange (lower) and blue (upper) color. The horizontal dashed line indicates the adjusted p-value cutoff (0.05). The vertical dashed lines represent a stoichiometry diff threshold abs(0.1).


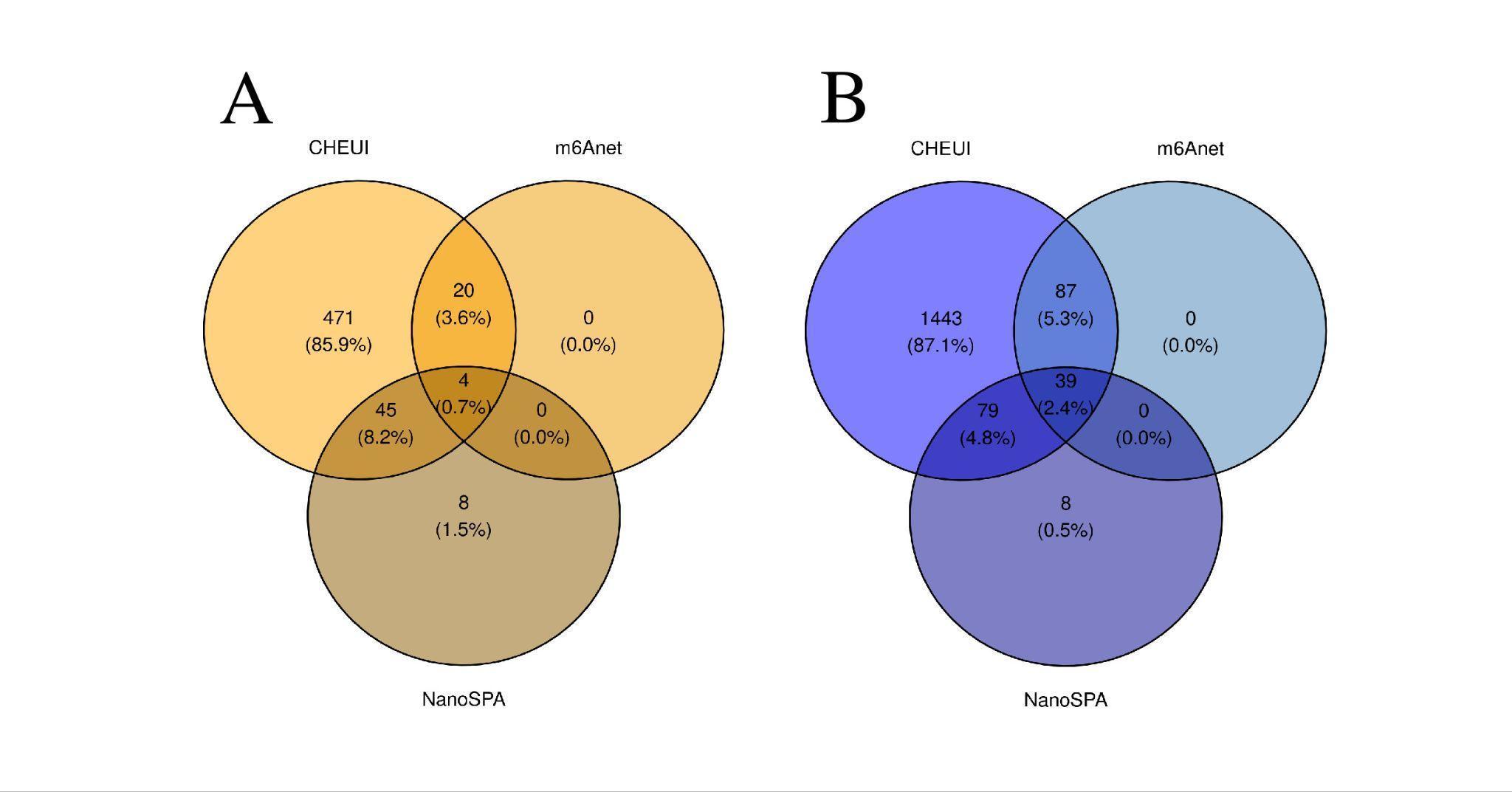


Figure S5. Overlap of m6A-modified transcripts in land and water. A,B - Venn diagrams illustrate the overlap between m6A-modified transcripts in: A - land and B - water.


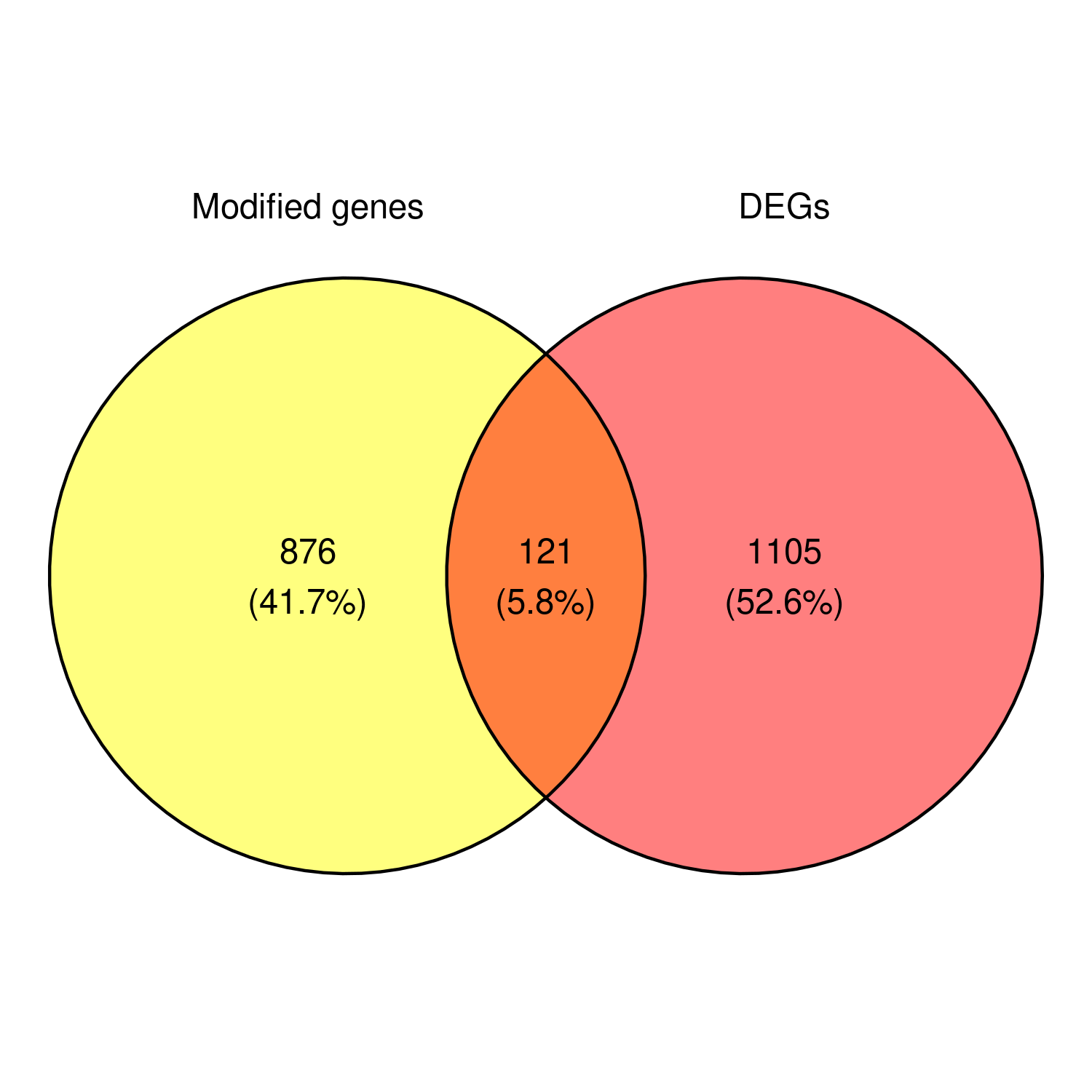


Figure S6. Venn diagram comparing modified genes and DEGs. Yellow indicates genes with modifications, red indicates DEGs, and orange represents genes that are both DEGs and have RNA modifications.
